# RAS specific protease induces irreversible growth arrest via p27 in several KRAS mutant colorectal cancer cell lines

**DOI:** 10.1038/s41598-021-97422-0

**Published:** 2021-09-09

**Authors:** Caleb K. Stubbs, Marco Biancucci, Vania Vidimar, Karla J. F. Satchell

**Affiliations:** 1 Department of Microbiology and Immunology, Feinberg School of Medicine, Northwestern University, Chicago, IL 60611 USA; 2 Present Address: GSK Vaccines, Rockville, MD 20850 USA; 3 Present Address: Merck Research Laboratories, Boston, MA 02115 USA

**Keywords:** Colon cancer, Checkpoints, Proteases, Recombinant protein therapy, Oncogenes

## Abstract

Ras-specific proteases to degrade RAS within cancer cells are under active development as an innovative strategy to treat tumorigenesis. The naturally occurring biological toxin effector called RAS/RAP1-specific endopeptidase (RRSP) is known to cleave all RAS within a cell, including HRAS, KRAS, NRAS and mutant KRAS G13D. Yet, our understanding of the mechanisms by which RRSP drives growth inhibition are unknown. Here, we demonstrate, using isogenic mouse fibroblasts expressing a single isoform of RAS or mutant KRAS, that RRSP equally inactivates all isoforms of RAS as well as the major oncogenic KRAS mutants. To investigate how RAS processing might lead to varying outcomes in cell fate within cancer cells, we tested RRSP against four colorectal cancer cell lines with a range of cell fates. While cell lines highly susceptible to RRSP (HCT116 and SW1463) undergo apoptosis, RRSP treatment of GP5d and SW620 cells induces G1 cell cycle arrest. In some cell lines, growth effects were dictated by rescued expression of the tumor suppressor protein p27 (Kip1). The ability of RRSP to irreversibly inhibit cancer cell growth highlights the antitumor potential of RRSP, and further warrants investigation as a potential anti-tumor therapeutic.

## Introduction

The oncoprotein Rat sarcoma GTPase (RAS) cycles between GTP-bound (active) and GDP-bound (inactive) states for activation of downstream effectors, each playing key roles in cell proliferation and survival^1,2^. This process is highly reliant on GTPase activating proteins (GAPs) and guanine exchange factors (GEFs) for hydrolysis of GTP and nucleotide exchange of GDP to GTP, respectively^3,4^. Upon growth receptor stimulation, activated RAS recruits downstream effectors, including Rapidly Accelerated Fibrosarcoma (RAF) kinase and phosphatidylinositol-3-kinase (PI3K). These effectors subsequently activate signaling pathways responsible for cell growth and survival, including the mitogen-activated kinase to extracellular signal-regulated kinase (ERK)^5^ signaling pathway and the protein kinase B (also known as AKT) to mammalian target of rapamycin (mTOR) pathway, respectively^6,7^.

Thirty percent of all human cancers contain mutations in *RAS*^2,8^*.* Mutant *RAS,* paired with loss of function in tumor suppressor genes such as *TP53* and *APC*, are sufficient to fully transform cells and drive tumorigenesis^8^. Nearly all *RAS* mutations occur as point mutations at Gly-12, Gly-13 or Gln-61, resulting in constitutive activation of RAS^2^. Among the major *RAS* isoforms (*HRAS*, *NRAS*, and *KRAS*), *KRAS* is the most frequently mutated isoform among all cancers (85%) followed by *NRAS* (11%) and *HRAS* (4%)^8^. *RAS* mutations are highly enriched specifically in three of the four most lethal cancers in the United States, including pancreatic adenocarcinoma (98%), colorectal adenocarcinoma (52%), and lung adenocarcinoma (32%)^2,8^.

Although numerous studies support the advantages of targeting RAS to treat cancer, it remains an unsolved challenge in the clinic^9–13^. Recent studies have taken advantage of biochemical properties of specific RAS mutants to develop selective small molecule inhibitors specific for highly oncogenic mutant forms of RAS. In particular, small molecules targeting KRAS G12C have been developed and are undergoing clinical trials^14–16^. Many of these agents have shown clinical success with one molecule receiving FDA accelerated approval earlier this year for treatment of KRAS G12C tumors in non-small cell lung carcinoma^17^. Despite this success, the strategy of selective inhibition has problems of being applicable to only a limited range of cancers integrated with personalized medicine and cannot be used to treat cancers that lack the specific mutation. To address this gap, new approaches are being developed to more broadly target RAS either with proteases that directly cleave RAS^18,19^ or with linkers that target RAS for cellular degradation^20–23^.

In line with this alternative strategy, our lab has identified a potent protease that cleaves RAS called the Ras/Rap1-specific endopeptidase (RRSP). RRSP is a small domain of a large toxin secreted by the bacterium *Vibrio vulnificus* during host infection. *V. vulnificus* delivers RRSP into intestinal epithelial cells during host infection, where it targets all RAS isoforms and close homolog Ras-related protein 1 (RAP1). Through RAS inactivation, this bacterium suppresses the host immune response, thereby aiding systemic dissemination of the bacterium^24,25^. Detailed structural and biochemical studies have shown that RRSP attacks the peptide bond between Tyr-32 and Asp-33 in the Switch I region of both RAS and RAP1^26^. As a result, RAS and RAP1 are unable to undergo GTP-GDP exchange or bind to their downstream effectors^27,28^. Recently, RRSP engineered as a chimeric toxin for in vivo delivery was shown to significantly reduce breast and colon tumor growth in xenograft mouse models^18^.

The potential applicability of RRSP to a broader range of cancers was screened using the standardized National Cancer Institute (NCI) cancer cell panel^29^. Fourteen of 60 cell lines were classified as highly susceptible with cells undergoing cytotoxic effects. However, 38/60 of cell lines showed growth inhibition, but not cytotoxicity. Only 8/60 showed low or no susceptibility with cell growing near normal rates, possibly due to lack of the receptor for the engineered chimeric toxin^18^. The observed wide range of cell fates highlights that the cellular responses to total RAS cleavage has the potential to be quite variable across cancer cell lines. Here, we investigate how RRSP processing affects cell signaling and demonstrate that cleaving total RAS can have a variable impact on cancer cell growth and survival. Specifically, we demonstrate that RRSP can disrupt colorectal cancer (CRC) cell growth through multiple mechanisms, including loss of cell viability, cell cycle arrest, and senescence.

## Results

### RRSP cleaves and inhibits proliferation in RAS wildtype and KRAS mutant cells

RRSP was previously shown to specifically cleave HRAS, NRAS, and KRAS when the proteins were ectopically expressed in HeLa cells and recombinant RRSP was shown to process purified KRAS G12D, G13D, and Q61R in biochemical assays^26^. To get an even broader sense of RRSP effectiveness across different isoforms and mutants of RAS, we tested RRSP against the ‘RAS-less’ mouse embryonic fibroblast (MEF) cell line panel developed by Drosten et al.^7^. These isogenic cell lines have endogenous *RAS* genetically deleted from their genome and a single allele of a human *RAS* gene is integrated to rescue growth. For delivery of RRSP into mouse cells, we used the anthrax toxin-based delivery system wherein the anthrax toxin lethal factor N-terminus was fused with RRSP (LF_N_RRSP) or LF_N_RRSP with a catalytically inactivating H4030A amino acid substitution (here after referred to as LF_N_RRSP*). Intracellular delivery of RRSP (previously known as DUF5) by anthrax toxin protective antigen (PA) was previously demonstrated in several mammalian and mouse cell lines^26,30,31^.

**Figure 1:**
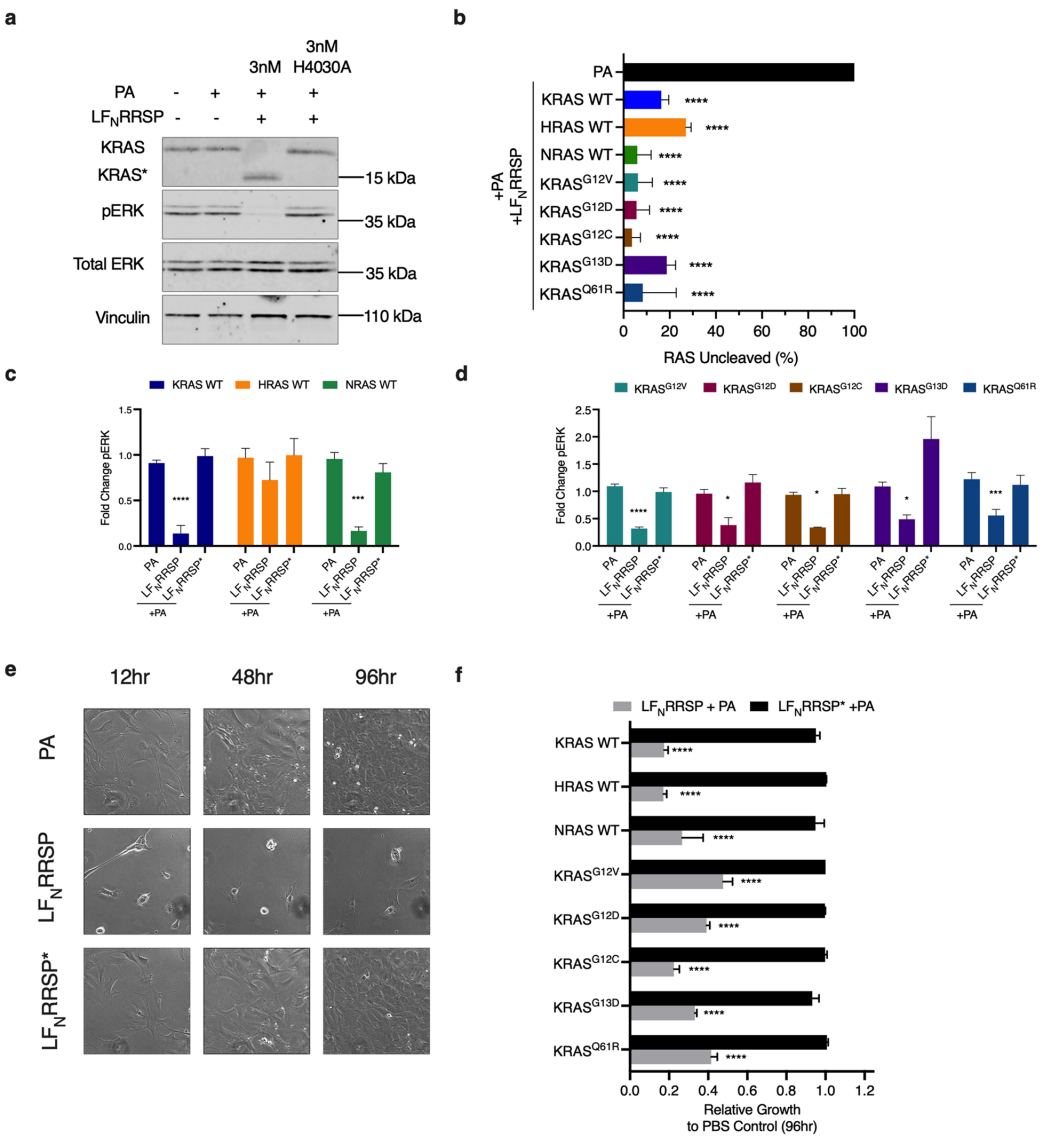
LF_N_RRSP cleaves and inhibits all RAS isoforms and KRAS oncogenic mutants in RAS-less MEFs. (**A**) Representative western blot analysis of LF_N_RRSP cleavage of RAS and inhibition of ERK in KRAS WT RAS-less MEFs after 24 h. Vinculin was used as a gel loading control. Protein detection for each immunoblot was analyzed on the same blot. Protein detection for each immunoblot was conducted on the same blot and cropped for each individual protein of interest. Full length blots can be found in Supplementary Fig. 7. (**B**) Densitometric analysis of total percent RAS in indicated RAS-less MEFs following LF_N_RRSP treatment for 24 h, *n* = 3. (**C,D**) Densitometric analysis of fold change in pERK compared to PBS control after 24 h for RAS-less MEF cell lines indicated; *n* = 3. (**E**) Brightfield images of KRAS WT RAS-less MEFs treated with either PA alone or in combination with LF_N_RRSP or LF_N_RRSP* at indicated timepoints. (**F**) Cell growth over time was monitored with time lapse video microscopy and quantified using Nikon Elements. Values shows relative growth inhibition in RAS-less MEFs compared to PA control at 96 h following treatment with either LF_N_RRSP or LF_N_RRSP*. Results for all panels are expressed as mean ± SEM of three independent experiments (*P < 0.05, **P < 0.01, ****P < 0.0001 versus PA control as determined through one-way ANOVA followed by Dunnett’s multiple comparison test).

In MEFs expressing human KRAS, HRAS, or NRAS, treatment with 3 nM LF_N_RRSP dramatically decreased intact full-length RAS levels with increased detection of cleaved RAS. For each isoform, LF_N_RRSP was found to cleave at least 80% of RAS after 24 h (Fig. 1A). As expected, controls treated with PA alone or in combination with catalytically inactive LF_N_RRSP* showed no change of intact RAS protein levels (Fig. 1A,B). We observed similar RRSP activity in MEF cell lines expressing oncogenic KRAS, including G12V, G12D, G12C, G13D, and Q61R. Amongst each of the mutant RAS alleles tested, we observed ~ 25% of total RAS remaining following LF_N_RRSP treatment, with no significant loss of RAS in cells treated with alone (Fig. 1B). The oncogenic RAS variants with the higher percentage of RAS remaining following LF_N_RRSP treatment were G13D and Q61R, although these differences were not statistically significant. Further, the total RAS remaining in each LF_N_RRSP-treated MEF cell line was not statistically significant between groups. In addition to cleavage of RAS, LF_N_RRSP treated cells showed significant decrease in phosphorylation of ERK when compared to cells treated with PA alone or with the catalytically inactive LF_N_RRSP* (Fig. 1C,D).

To test the impact of processing of different RAS isoforms on cell proliferation, RRSP-treated cells were tracked using time lapse imaging for four days. At early timepoints following treatment, LF_N_RRSP-induced a severe cell rounding that was not observed in PA alone and LF_N_RRSP* control treated cells (Fig. 1E). This phenotype is consistent with previous studies with RRSP and is possibly linked to cleavage of RAP1, which regulates cytoskeletal dynamics^26,30,32^. LF_N_RRSP treated cells showed reduced confluency at both 48 and 96 h (Fig. 1E) and continuous treatment for 96 h resulted in at least at least a 60% reduction in confluency for all RAS-less MEF cell lines compared to cells treated with either PA only or LF_N_RRSP* mutant controls (Fig. 1F, Supplementary Fig. 1).

Altogether, these results in MEFs demonstrate that RRSP is equally able to cleave all isoforms of RAS and mutant KRAS to inhibit both ERK phosphorylation and cell proliferation in a defined system. Thus, the KRAS mutation does not likely solely account for differences in cancer cell fate upon treatment with RRSP. Instead, the differences more likely depend on processes downstream of RAS processing that could vary in different cell lines.

### RRSP inhibits proliferation and pERK activation in CRC cell lines

**Figure 2:**
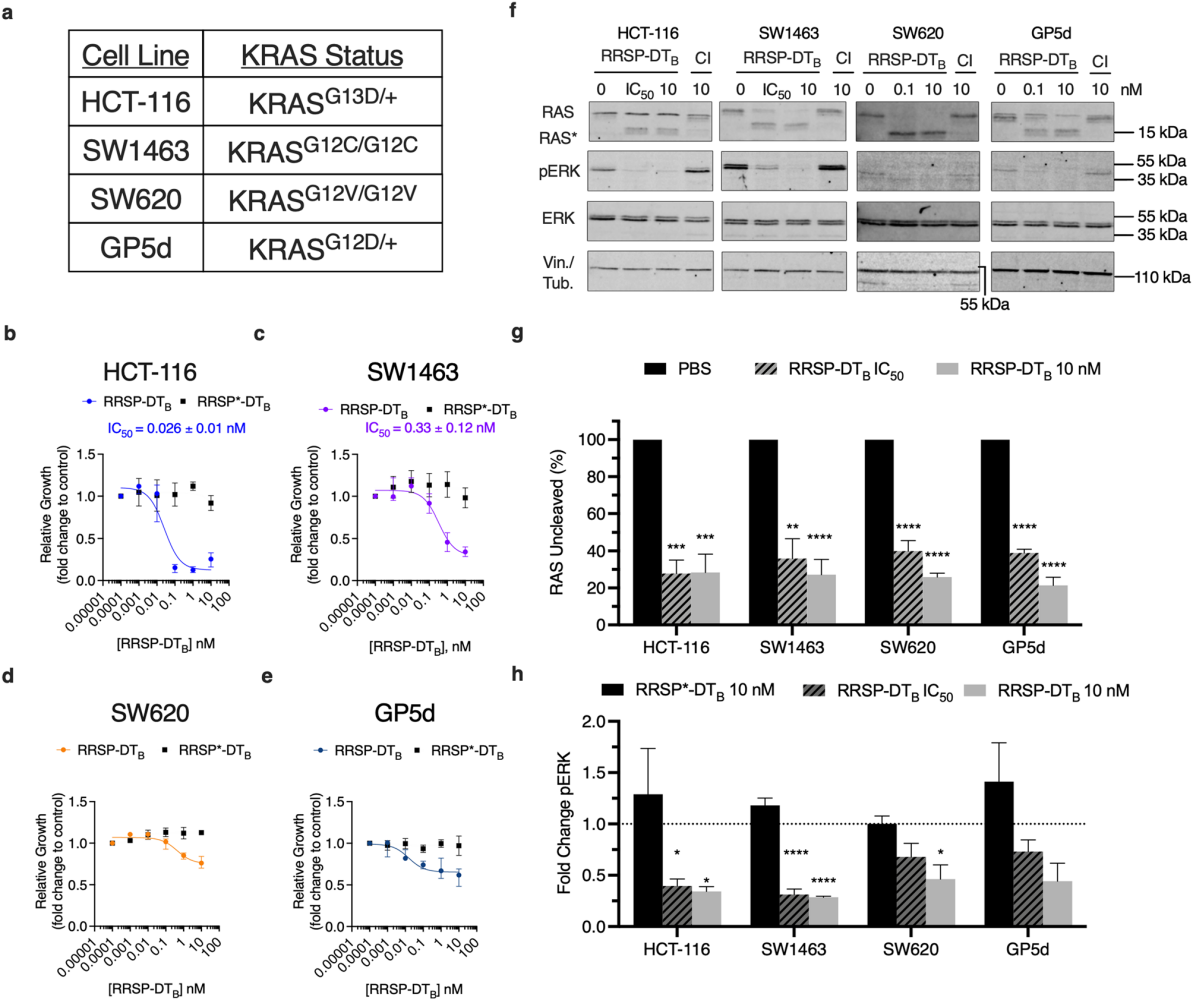
RRSP-DT_B_ growth inhibition in CRC cell lines. (**A**) Cell line panel of KRAS mutant CRC cells used in this study. (**B–E**) Cell growth over time was monitored by time lapse video microscopy and cell confluency was quantified using ImageJ. Fitted dose response curve of RRSP-DT_B_ in CRC cell lines show relative growth compared to PBS control after 24 h. Results are displayed as mean ± SEM, *n* = 4. (**F**) Representative western blot analysis of RAS cleavage and ERK inhibition in CRC cell lines treated with either RRSP-DT_B_ or catalytically inactive mutant (labeled by CI) after 24 h. All concentrations are expressed in nanomolar. In all cell lines, vinculin was used as gel loading control except SW620 cells in which αTubulin was used. Protein detection for each immunoblot was conducted on the same blot and cropped for each individual protein of interest. Full length blots can be found in Supplementary Fig. 8. (**G,H**) Densitometric analysis of fold change in percent total RAS and pERK compared to PBS control after 24 h in CRC cell lines; *n* = 3. IC_50_ concentrations for HCT-116 and SW1463 can be found in Figs. 2B and 2C. For cell lines where IC_50_ values could not be extrapolated (SW620 and GP5d) RRSP-DT_B_ was used at 0.1 nM. Results are expressed as means ± SEM of three independent experiments (*P < 0.05, **P < 0.01, ****P < 0.0001 versus PBS control as determined through one-way ANOVA followed by Dunnett’s multiple comparison test).

To probe the effect of processing of RAS on downstream signaling, we focused on four KRAS mutant CRC cell lines, each harboring different allelic mutations in *KRAS* (Fig. 2A). Due to problems with variable expression of the anthrax toxin receptor on the selected human cancer cells, we switched to a recently described, highly potent RRSP chimeric toxin wherein RRSP is tethered to the translocation B fragment of diphtheria toxin (RRSP-DT_B_)^18^. Similar to the anthrax toxin system, RRSP-DT_B_ binds to a human receptor (heparin binding epidermal growth factor-like growth factor (HB-EGF)), is endocytosed, and translocated into the cytosol across the vacuolar membrane. Expression of HB-EGF receptor was found to be similar between the selected CRC cell lines (Supplementary Fig. 2, Supplementary Fig. 10).

To examine RRSP growth sensitivities between the CRC cell lines, cells were treated with increasing concentration of RRSP-DT_B_ or with catalytically inactive RRSP-DT_B_ (RRSP*-DT_B_) and growth inhibition was monitored. HCT-116 cells showed the greatest impact of RRSP-DT_B_ on cell confluency and the lowest IC_50_ (Fig. 2B, Supplementary Fig. 2), consistent with prior data using different methods that HCT-116 cells are highly susceptible to RRSP^18^. Cells treated with catalytically inactive RRSP*-DT_B_ showed no difference, confirming the sensitivity was due to processing of RAS (Fig. 2B, Supplementary Fig. 2). SW1463 cells were also highly susceptible to RRSP-DT_B_ but with a 12-fold higher IC_50_ compared to HCT-116 (Fig. 2C, Supplementary Fig. 2). Cell line SW620 was less susceptible to RRSP-DT_B_ after 24 h with about a 40% growth inhibition compared with control at the highest dose tested of 10 nM. This result using a different method is consistent with prior results^18^, which categorized SW620 as responding to RRSP by growth inhibition, although the percent inhibition here was less due to the earlier time point used for comparison. Cell line GP5d was also less susceptible to RRSP, but also showed growth inhibition when compared to cells treated with the control (Fig. 2D,E, Supplementary Fig. 2). Across all of the cell lines, at least 80% of total RAS was cleaved by RRSP (Fig. 2F,G). In addition, phosphorylation of ERK was significantly reduced compared to respective RRSP*-DT_B_ treated samples (Fig. 2F,H). We did observe some variability in detection of uncleaved RAS between cell lines, which can be attributed in part to cells sensing depleted pools of RAS and therefore upregulating expression. This is best observed in HCT-116 cells treated with RRSP-DT_B_ where total uncleaved RAS protein levels increase above the levels of PBS control even as cleaved RAS accumulates (Fig. 2F).

**Figure 3:**
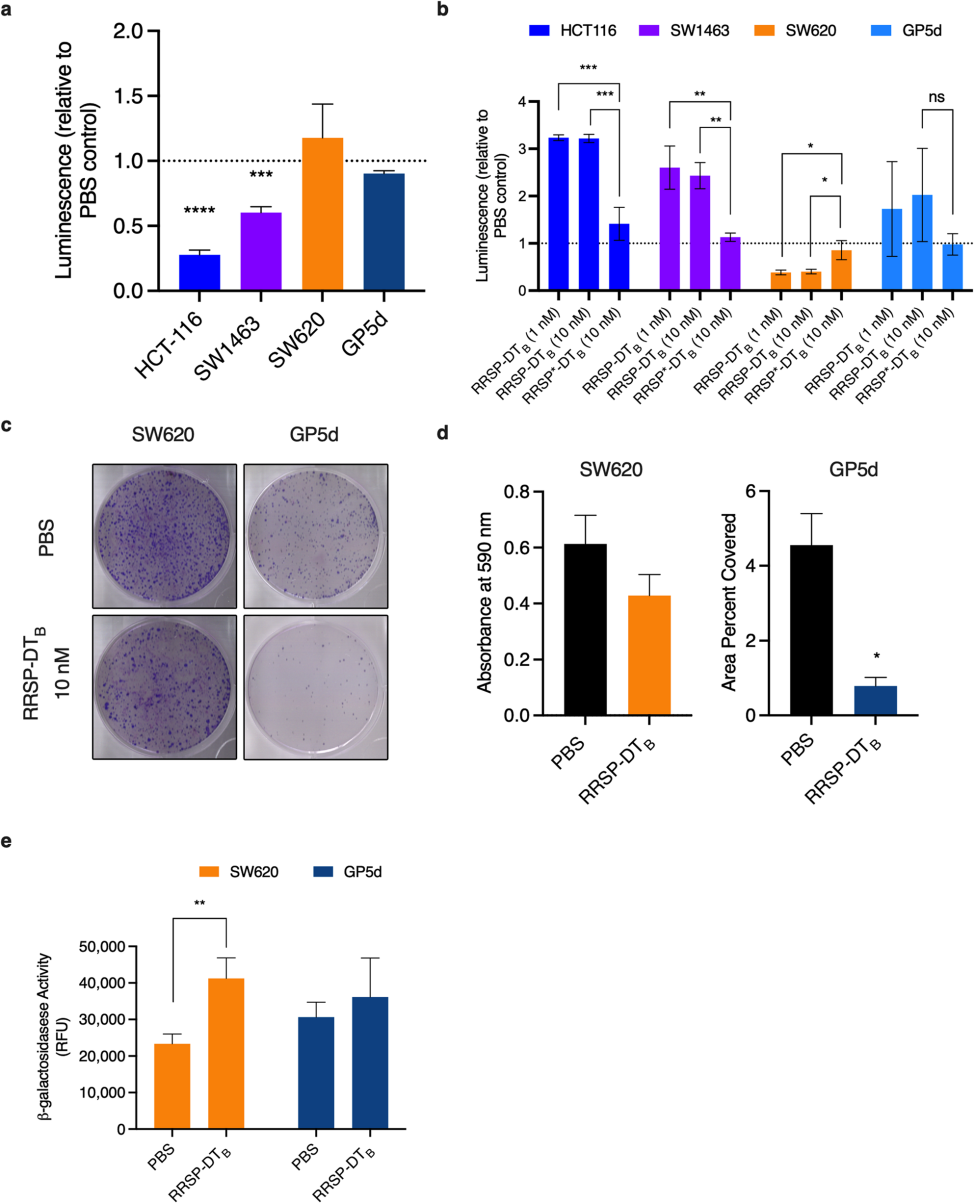
RRSP-DT_B_ decreases cell viability in highly sensitive cell lines but causes irreversible growth inhibition in lower susceptible cell lines. (**A**) Relative cellular metabolic activity quantified using CellTiterGlo assay after 72-h treatment with 10 nM RRSP-DT_B_ compared to PBS control in CRC cell lines. (**B**) Relative apoptosis quantified using Caspase-Glo 3/7 assay after 48-h treatment with either 1 or 10 nM RRSP-DT_B_ compared to PBS control in CRC cell lines. (**C**) Representative images of crystal violet-stained colonies from RRSP less sensitive cell lines pretreated with 10 nM RRSP-DT_B_ for 48 h and replated at low seeding density to form colonies over 14 days. (**D**) Quantitative analysis of crystal-violet stained colonies from less sensitive RRSP cell lines from three independent experiments. Results are expressed as means ± SEM of three independent experiments (**E**) Measured cell senescence activity in RRSP less sensitive cell lines treated with 10 nM RRSP-DT_B_ for 48 h then incubated with SA-ß-Gal Substrate for 1 h at 37ºC, *n* = 3. All results described above are expressed as mean ± SEM of three independent experiments (*P < 0.05, **P < 0.01, ****P < 0.0001, ns = not significant versus PBS control as determined through one-way ANOVA followed by Dunnett’s multiple comparison test).

The differences in growth following RRSP treatment further impacted long term survival. Using ATP as an indicator of cell viability, the CellTiterGlo Assay can quantitatively measure the presence of metabolically active cells through detection of luminescence signal, even if the cells fail to proliferate. In highly susceptible cell lines HCT-116 and SW1463 cells, treatment with RRSP-DT_B_ resulted in significantly decreased luminescence compared to mock treated controls after 72 h (Fig. 3A). By contrast, GP5d and SW620 showed no difference in relative ATP levels after 72 h.

To further understand the survival differences between RRSP-treated cell lines, we used the Caspase-Glo 3/7 Assay, which quantitatively measures caspase-3/7 activity using luminogenic caspase-3/7 substrate to indicate onset of apoptosis. In highly susceptible cell lines HCT-116 and SW1463, treatment with RRSP at both low (1 nM) and high (10 nM) concentrations of RRSP-DT_B_ significantly increased luminescence compared to mock-treated controls after 48 h, suggesting onset of apoptosis (Fig. 3B). This was not the case for GP5d cells where the signal was highly variable across replicate samples and even decreased in response to RRSP-DT_B_ treatment. For SW620 cells, we observed RRSP-DT_B_ treatment significantly decreased luminescence compared to mock-treated control suggesting a suppression of apoptosis. When treated for 48 h and reseeded at low cell densities SW620 and GP5d both showed a decrease in colony formation, suggesting that RRSP can induce a permanent non-proliferative state, even as cells maintain metabolic activity (Fig. 3C,D). SW620 cells also showed significant increase activity of the enzyme β-galactosidase, a marker of senescence. This would support our earlier observation in which apoptosis was suppressed in RRSP-DT_B_-treated SW620 cells since senescence is known to counteract apoptosis pathway activation^33^. However, β-galactosidase activity of treated GP5d cells remained unchanged (Fig. 3E). Altogether, these data demonstrate that RRSP activity results in induction of apoptosis in highly susceptible cell lines while, in less sensitive lines, the cells remain metabolically active, but are unable to proliferate and, in some cases, enter into senescence.

### RAS cleavage can induce upregulation of cyclin-dependent kinase inhibitor p27 and hypo-phosphorylation of RB

**Figure 4:**
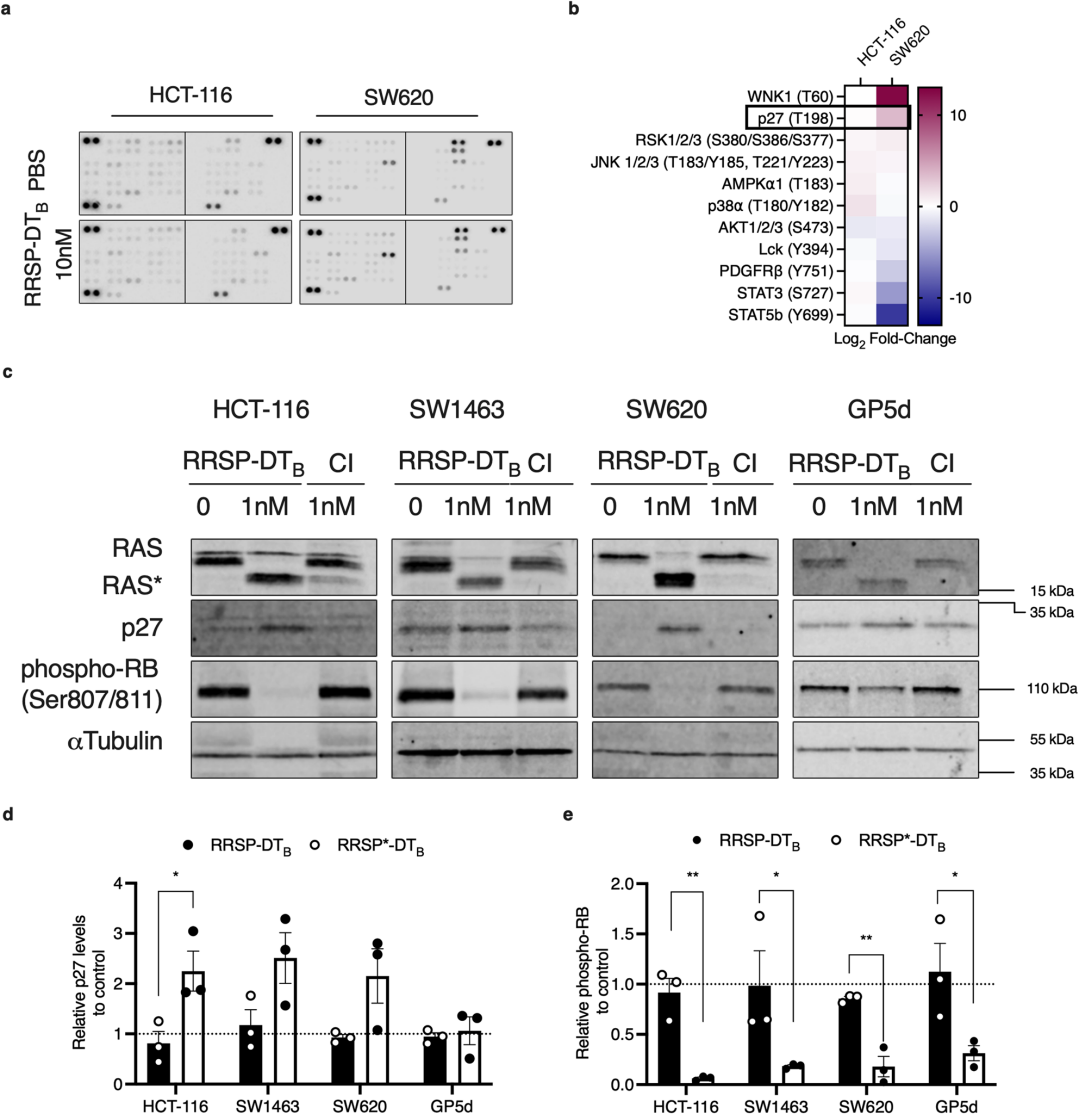
RRSP-DT_B_ cleavage of RAS induces p27 expression in CRC cell lines. (**A**) Human phospho-kinase array blots of HCT-116 and SW620 cells treated with either PBS or RRSP-DT_B_ (10 nM) for 24 h. (**B**) Densitometric analysis kinase array depicted through a heatmap of relative phosphorylated proteins levels in response to RRSP-DT_B_ compared to PBS control in HCT-116 and SW620 cells, *n* = 1. (**C**) Representative western blot images of p27 and phosphor-RB expression in CRC cell lines treated with either RRSP-DT_B_ or RRSP*-DT_B_ for 24 h. Protein detection for each immunoblot was conducted on the same blot and cropped for each individual protein of interest. Full length blots can be found in Supplementary Fig. 9. (**D,E)** Densitometric analysis of fold change in p27 and phospho-RB compared to PBS after 24 h in RRSP-DT_B_ treated CRC cell lines; *n* = 3. αTubulin was used as gel loading control. Results are expressed as mean ± SEM of three independent experiments (*P < 0.05, ** < 0.01, **** < 0.0001 versus PBS control as determined through one-way ANOVA followed by Dunnett’s multiple comparison test).

We next took advantage of the unique cell line specific effects on cell growth and survival to better understand the underlying mechanisms regulating cell fate following RAS inhibition. Cell lysates from treated or untreated HCT-116 (highly sensitive) and SW620 (less sensitive) were incubated overnight with nitrocellulose membranes containing capture antibodies towards 43 different phosphorylated proteins (Fig. 4A). For RRSP-treated HCT-116 cells, there was increased phosphorylation observed for cell stress proteins such as p38α, p90 ribosomal S6 kinase (RSK1/2/3), and Jun-activated kinase (JNK) (Fig. 4B, Supplementary Fig. 3). In addition, RRSP treatment increased phosphorylation of several Signal Transducer and Activator of Transcription (STAT) transcription factors. By contrast, the less responsive SW620 cells showed decreased phosphorylation of several STAT proteins (Fig. 4B, Supplementary Fig. 3). We also observed a significant fold increase in With No K(lysine)-1 (WNK1) kinase at Thr-60. This kinase is phosphorylated by AKT in HEK293 cells and is best known for regulating ion transport across membranes^34^. However, phosphorylation of Thr-60 has no effect on its kinase activity or its cellular localization^35^. Because RRSP decreases AKT activation (Fig. 4B), it is unlikely that WNK1 Thr-60 phosphorylation is involved in the growth differences we observe between cell lines.

Thus, we focused on the large fold-change difference observed in phosphorylation at Thr-198 of the cyclin-dependent kinase inhibitor p27 (also known as Kip1) (Fig. 4B). While HCT-116 cells showed no significant change in p27 phosphorylation in the screen, SW620 showed a threefold increase in p27 phosphorylation. RAS is known to regulate critical components involved in cell cycle. RAS activation is directly linked to hyper-phosphorylation of retinoblastoma protein (RB), thereby relieving its repression of E2F transcription factors, allowing transcription of G1 promoting genes, and promoting the cell cycle to progress from G1 to S phase^36^. Previous studies have established that phosphorylation of p27 at Thr-198 is critical for stabilizing p27 expression by preventing ubiquitin-dependent degradation^37^. In fact, aberrant RAS activity in cancer cells causes p27 post-translational downregulation through both ERK and AKT^5,38,39^. These data support that inhibition of RAS by RRSP could lead to downstream rescue expression of p27 expression in the SW620 cells, thereby slowing reversing the hyper-phosphorylation of RB.

To test this possibility, all four cancer cell lines were treated with a sublethal dose of RRSP-DT_B_. The treatment increased p27 protein levels in HCT-116, SW620, and SW1463 cells, while in GP5d cells levels remained unchanged (Fig. 4C,D). Concomitant with increased abundance of p27, all cell lines showed a significant decrease in RB phosphorylation at Ser-807/Ser-811 (Fig. 4C,E). Unfortunately, total RB was undetectable using commercially available antibodies. To be confident that RB hypo-phosphorylation was not due to low RB expression, we transiently expressed green-fluorescent protein (GFP)-tagged RB in HCT-116 cells (Supplementary Fig. 4, Supplementary Fig. 11). In GFP-RB expressing cells treated with RRSP-DT_B_, hypo-phosphorylation of RB protein compared to PBS and RRSP*-DT_B_ controls was observed (Supplementary Fig. 4). Protein levels of total GFP-RB were decreased upon treatment with RRSP-DT_B._ This result was expected and is consistent with a role of p27 in degradation of RB protein to promote growth arrest^40^.

Unexpectedly, hypo-phosphorylation of RB was also observed in GP5d cells despite showing no change in the expression or phosphorylation of p27 (Fig. 4D,E). The cyclin-dependent kinase inhibitor, p21, also plays a critical role in RB regulation. However, there was also no change in p21 protein levels in RRSP-treated GP5d cells (Supplementary Fig. 4, Supplementary Fig. 12).

### RRSP induces G1 phase cycle arrest

**Figure 5:**
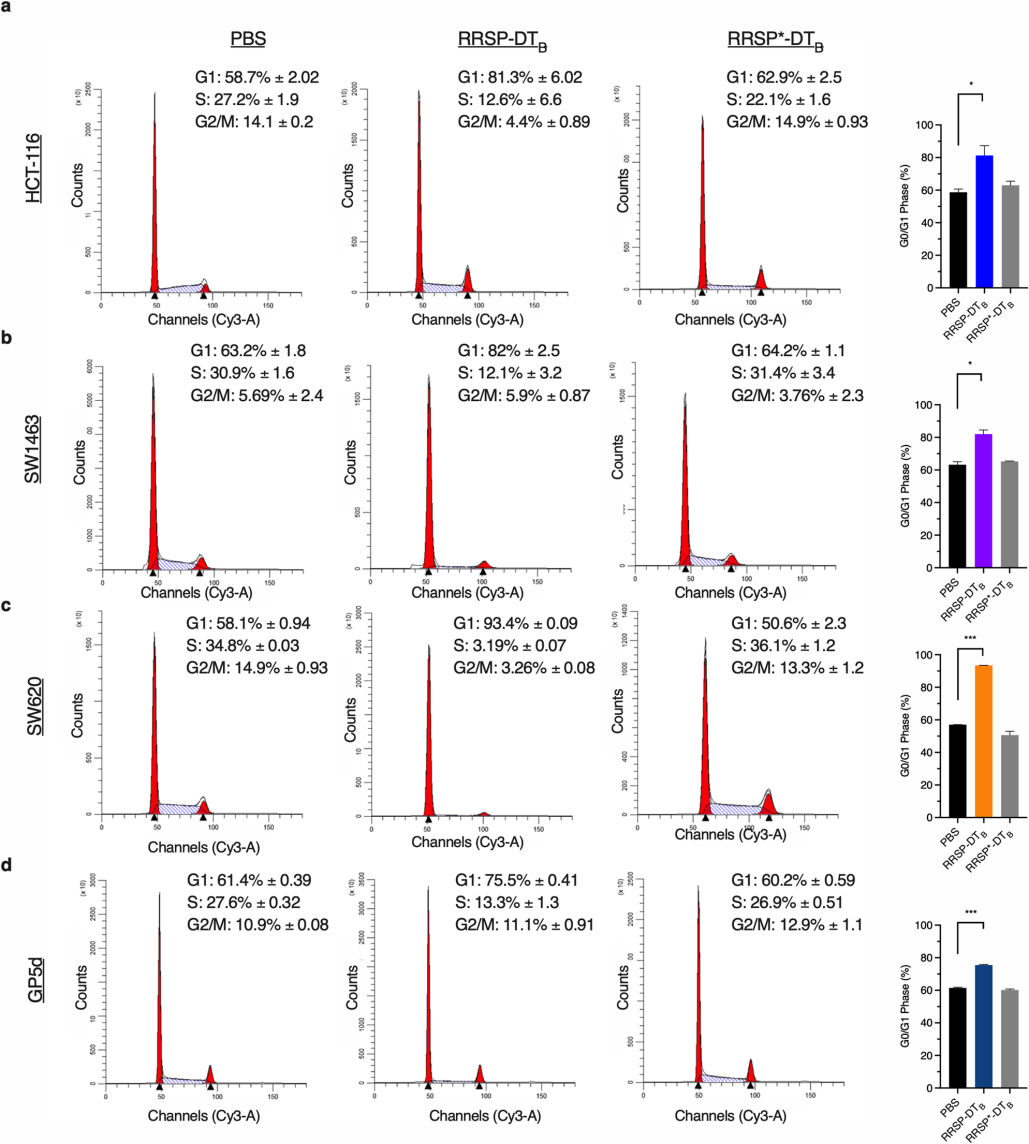
RRSP-DT_B_ induces G1 cell cycle arrest in CRC cell lines. (**A–D**) Cell cycle flow cytometry analysis of CRC cell lines treated with either PBS, RRSP-DT_B_ or RRSP*-DT_B_ (1 nM) for 24 h. Percentage of cells in each phase are an average of three independent experiments. Bar graphs depict percentage of cells in G1 phase for each treated sample; *n* = 3. Results are expressed as mean ± SEM of three independent experiments (*P < 0.05, **P < 0.01, ****P < 0.0001 versus PA control as determined through one-way ANOVA followed by Dunnett’s multiple comparison test).

Elevated p27 protein expression in combination with hypo-phosphorylation of RB suggested that RRSP treatment induces cell cycle arrest. Under normal conditions, p27 regulates G1 checkpoint during the cell cycle by preventing entry into S phase through inhibition of CDK2^41,42^. To test if RRSP-DT_B_ treatment induces cell cycle arrest, cell lines were treated for 24 h and the percentage of cells in G1, S, or G2/M phase was monitored. All cell lines that showed reduced RB phosphorylation had significant population of cells locked in the G1 state compared to PBS and RRSP*-DT_B_ treated samples (Fig. 5, Supplementary Fig. 6). The most dramatic increase in G1 arrest was seen in SW620 cells, where nearly 100% of cells remained in the G0/G1 phase following RRSP-DT_B_ treatment (Fig. 5C). This G1 cell arrest was dependent on the RAS processing activity of RRSP as the catalytically inactive mutant RRSP*-DT_B_ did not induce the cell cycle arrest (Fig. 5A–D). Together, these data illustrate that RRSP cleavage of RAS can induce growth inhibition through inducing cell cycle arrest, after which some cell lines progress to cytotoxic death.

## Discussion

It has been over 30 years since the discovery of the importance of RAS for driving tumorigenesis in cancer. Lung, pancreatic, and colorectal cancers remain being the most lethal cancers in the United States with high mutation rates in KRAS, the most commonly mutated isoform. Despite the significant amount of research being conducted on RAS, it still remains a challenging target in the field. Small molecules directed to specific RAS mutants, specially KRAS G12C, have shown promising results in clinical trials^43^, but will only benefit a small subset of patients. Our lab has discovered RRSP as a potent, site specific inhibitor of RAS capable of inhibiting all RAS isoforms simultaneously along with downstream activation. RRSP antitumorigenic effects are well demonstrated in vivo with xenograft models for both breast and colon cancers, wherein tumor growth was stunted and, in some cases, showed regression^18^. Evidence for RRSP as a therapeutic inhibitor of RAS is sufficient, however the mechanism by which RRSP mediates growth inhibition has been an outstanding question. In this study, we examined the signaling consequences of cleavage of all RAS in several CRC cell lines and its downstream implications on cell proliferation and survival. The goal of the study was to understand the mechanism of growth inhibition in response to RRSP, in the absence of cytotoxicity.

First, we examined whether RRSP was a suitable inhibitor across RAS variants. Using the isogenic ‘RAS-less’ MEF model, we demonstrated that all three major RAS isoforms and frequently observed KRAS mutants were equivalent substrates for RRSP. Loss of RAS resulted in reduced ERK activation, which as expected, negatively affected proliferation. Most importantly, in this isogenic study, we observed no significant differences in RAS cleavage between wildtype isoforms and KRAS mutants. RAS-less MEFs are useful for studying isolated biochemical properties and signaling of specific oncogenic RAS in a cellular context making it an excellent model to study RRSP targeting of mutant RAS. One of the unavoidable disadvantages of this model is the number of integration sites that vary between RAS-less MEF cell lines. For some RAS cell lines a single gene integration could not rescue proliferation and required multiple integrations for stable clonal populations. As a result in the context of RRSP, expression of multiple genes of a single RAS allele may lead to an underestimation of RRSP effectiveness in cleaving RAS.

We next examined RRSP effectiveness in four CRC cell lines, which displayed variations in susceptibility to RRSP. Two of the cell lines with the greatest RRSP growth sensitivity, HCT-116 and SW1463, had dramatically lower metabolic activity and induction of apoptosis compared to controls. Interestingly, GP5d and SW620 retained normal metabolic activity, yet showed significant inability to form colonies following RRSP treatment, mimicking a senescent-like phenotype. This observation is consistent with prior data for SW620 showing a reduction in cellular material stained with sulforhodamine B after 48 h of treatment at 13.5 nM RRSP, but RRSP was not cytotoxic^18^. A logical hypothesis is that GP5d and SW620 cells have elevated pERK activity and therefore are less susceptible to RRSP. However, our data show the opposite in that moderately susceptible cell lines have significant decreases in pERK and RAS following RRSP treatment. This result would suggest that inhibition of pERK activity is not a predictor of susceptibility to RRSP in many cell lines. Similar results have previously demonstrated that varying growth sensitivities to pharmacological inhibition of mitogen-activated protein kinase kinases (MEKs) do not correlate with pERK activity in KRAS and BRAF mutant CRC cell lines^44,45^. Further investigation of RRSP in the context of KRAS mutants expressing low levels of pERK would elucidate mechanisms regulating cell fate signals between cell lines.

Mechanisms that link RAS and the cell cycle have been well examined. In quiescent cells, p27 is highly expressed in order to inhibit CDKs activity and to suppress RB phosphorylation^41,42^. Upon mitogen stimulation, RAS activation suppresses p27 protein expression through post-translational modifications that signal for its ubiquitin-mediated degradation^5,38,39^. In RAS-driven human cancers, low levels of p27 are frequently observed. We demonstrated that the growth inhibition in HCT-116, SW1463, and SW620 is the result of G1 cell cycle arrest through the upregulation of p27. These data suggest that RAS cleavage in certain CRC cell lines induces p27 upregulation, leading to a cell cycle arrest state that can induce apoptosis at later timepoints. Transient overexpression of p27 is known to then induce cell cycle arrest and later apoptosis^46,47^. Although in our studies, only the highly susceptible cell lines showed decreases in viability and induction of apoptosis, whereas SW620 retained metabolic activity and undergoes cell cycle arrest. It is important to note that a prolonged cell cycle arrest through p27 induces a persistent cell cycle arrest through induce senescence^48^. We observe this phenotype in SW620 cells in which RRSP treatment induced a senescent-like phenotype.

Unexpectedly, GP5d cells did not show upregulation of p27 or p21, although RB was hypo-phosphorylated and cells initiated G1 cell cycle arrest. These data reveal there must be other pathways that drive growth inhibition following RAS cleavage. In fact, RRSP from the insect pathogen *Photorhabdus asymbiotica*, which is identical to RRSP, also cleaves RAS^26^ and was recently reported to induce G1 cell cycle arrest^49^. The proposed mechanism did not depend on RAS processing, and instead involved RRSP directly binding to CDK1 when it is overexpressed, essentially functioning as a protein trap. Thus, the multi-domain RRSP may possess at least two mechanisms to inhibit the cell cycle, one by restoring p27 downstream of RAS processing and another by directly binding to CDK1. Notably, because low p27 expression levels have been correlated with poor survival in patients with different types of cancer including colon, the ability of RRSP to restore p27 expression and to initiate cell cycle arrest could have important implications for the treatment of tumors with aberrant RAS signaling.

In summary, a critical finding of this study was that processing of RAS does not result in a single easily tractable cell fate in all cancer cells, but drives a suite of alternative overlapping outcomes that can include cytotoxicity, inhibition of cell cycle and senescence. The differences are not driven by the nature of *KRAS* oncogenic mutation as all mutant forms of KRAS were susceptible to RRSP-driven RAS processing in an isogenic system and RAS was processed in all the CRC cell lines. The impact of RAS processing is thus linked to cancer cell differences in signal pathways downstream of RAS. A limitation of this study is that only four lines were assessed, suggesting that the array of variable outcomes could increase as additional cell lines are considered in the future. However, the key finding here is that, despite differences in the mechanisms underlying RRSP susceptibility, all cells tested were ultimately susceptible and all led to reduced cell viability, growth, and/or proliferation. Thus, RAS processing or degradation has great promise as a potential broadly applicable therapy against colon cancer.

## Materials and methods

### Cell lines

‘RAS-less’ mouse embryonic fibroblast (MEF) cells were provided by the NCI RAS Initiative at Frederick National Laboratory for Cancer Research (FNLCR). HCT-116 cells were purchased from the American Type Culture Collection. SW1463, GP5d, and SW620 were provided by the NCI. Each cell line was validated by the Northwestern University Sequencing Core by Short Tandem Repeat profiling.

All cells were cultured at 37 °C and 5% CO_2_ atmosphere. HCT-116, SW1463, GP5d, SW620 cells were cultured in Dulbecco’s Modified Eagle Medium (DMEM)-F12 with Glutamax (Gibco) containing 10% fetal bovine serum (FBS; Gemini Bio) and 1% penicillin/streptomycin (P/S; Invitrogen). All MEF cells, except for HRAS RAS-less MEFs, were cultured in DMEM (Gibco) with 10% FBS,1% P/S, and 4 µg/ml of blasticidin (ThermoFisher Scientific). HRAS RAS-less MEFs was cultured in 2.5 µg/ml of puromycin (ThermoFisher Scientific).

### Antibodies

Anti-RAS monoclonal antibody recognizing G-domain of all major RAS isoforms was purified from a hybridoma cell line provided by FNLCR and used at 1:2000 dilution as previously described^18^. Other commercially available primary antibodies used were: anti–Phospho-p44/42 MAPK (phosphorylated ERK1/2, Thr202/Tyr204, Cell Signaling Technology #4377), anti-p44/42 MAPK (ERK1/2, Cell Signaling Technology #4696), anti-HB-EGF (R&D Systems, #AF-259-NA;), anti-p27^Kip1^ XP (Cell Signaling Technology #3686), Phospho-RB (Ser807/Ser811, Cell Signaling Technology #8516), anti-p21^WAF/Cip1^ (Cell Signaling Technology #2947T), anti-α-Tubulin (Cell Signaling Technology #2144), and anti-vinculin (Cell Signaling Technology #13901). Fluorescently-labeled secondary antibodies obtained from LI-COR Biosciences and used at 1:10,000 dilution were: IRDye 680RD goat anti-mouse (926-68070), IRDye 800CW goat anti-rabbit (925-322211), and IRDye 800CW donkey anti-goat (925-32214). Western blot images were acquired using an Odyssey Infrared Imaging System (LI-COR Biosciences) and quantified by densitometry using NIH ImageJ software.

### pRB-GFP transfection

Plasmid RB-GFP FL for expression of GFP-tagged RB was obtained from Addgene (Catalog #16004). For ectopic gene expression, cell lines were transfected using FuGene HD (Promega) according to the manufacturer’s protocols. GFP fluorescence was analyzed using EVOS XL Core imaging system.

### Western blotting

Cells were washed in PBS and then resuspended in lysis buffer [150 mM NaCl, 20 mM Tris (pH 7.5), 1% Triton X-100, and Pierce Protease phosphatase inhibitor (Sigma-Aldrich)]. Lysates were incubated for 15 min on ice and centrifuged at 20,000 × *g* at 4 °C for 15 min. The concentration of protein in the collected supernatant fluid was determined using the bicinchoninic acid (BCA) assay (ThermoFisher Scientific, no. 23227). Samples were boiled at 95 °C in Laemmli SDS loading buffer for 10 min and protein was separated on either 15 or 18% SDS–polyacrylamide gels. Proteins were transferred to nitrocellulose membranes and blocked in Tris-buffered saline (TBS) [10 mM Tris (pH 7.4) and 150 mM NaCl] with 5% (w/v) milk for 1 h. Membranes were washed with TBS and then incubated in indicated primary antibodies in TBS with 5% (w/v) Fraction V bovine serum album (Fisher BioReagents #194850) overnight at 4 °C. Total percentage RAS was calculated using the following equation: % Total RAS = uncleaved RAS band / (RAS uncleaved band / RAS cleaved band) X 100.

### Purification and intoxication of LF_N_RRSP in MEFs

Recombinant LF_N_RRSP and LF_N_RRSP* were expressed in *Escherichia coli* BL21(DE3) and purified over a HisTrap FF nickel affinity column followed by Superdex 75 size exclusion chromatography using the ÄKTA protein purifier purification system (GE Healthcare), as previously described^30^. For intoxication, MEFs were seeded in 6-well plates at 3 × 10^5^ cells per well for 1 h, after which medium was replaced with fresh medium containing with 7 nM Protective antigen (PA) alone (List Labs, #171E) or in the presence of 3 nM LF_N_RRSP/ LF_N_RRSP^H4030A^ and incubated at indicated timepoints at 37 °C in the presence of 5% CO_2_.

### Purification and intoxication of RRSP-DT_B_ in CRC cell lines

Recombinant RRSP-DT_B_ and RRSP*-DT_B_ were expressed in *E. coli* BL21(DE3) and purified over a HisTrap FF nickel affinity column as previously described^18^. Eluted fractions were loaded onto a gravity column containing Strep-Tactin Superflow high capacity resin, followed by SUMO-tag removal and size exclusion purification over a Superdex 75 column using ÄKTA protein purifier purification system as previously described^18^. For intoxication, CRC cell lines were seeded in 6-well plates (~ 70% confluency) overnight, after which medium was replaced with fresh medium containing either RRSP-DT_B_ or RRSP*-DT_B_ and incubated at indicated timepoints at 37 °C in the presence of 5% CO_2_.

### Time-lapse video microscopy

For RAS-less MEFs (6 × 10^3^ cells per well) were cultured in 96-well clear bottom white plates in corresponding complete growth medium and treated after 4 h with RRSP-DT_B_ or RRSP*-DT_B_. Colorectal cancer cell lines were plated at ~ 80% confluency and cultured in 96-well clear bottom white plates. Complete growth medium with RRSP-DT_B_ or RRSP*-DT_B_ was added after overnight cell attachment. All cells were cultured were in Nikon Biostation CT and images were taken at indicated timepoints. Cell confluency was quantified using Nikon Elements software. IC_50_ concentrations were calculated using log(inhibitor) vs. response variable slope (four parameters) function in Graphpad Prism 8.

### Cell viability, apoptosis, and cell survival assays

For cell viability assay CRC cell lines were seeded in 96-well clear bottom white plates at ~ 80% confluency. Complete growth medium with RRSP-DT_B_ or RRSP*-DT_B_ was added after overnight cell attachment. After 72 h, CellTiter-Glo (Promega) reagent was added to each well and luminescence was detected using Tecan Safire2 plate reader. For apoptosis assay, CRC cell lines were seeded at 10,000 cells per well in a 96-well clear bottom white plate. Complete growth medium with RRSP-DT_B_ or RRSP*-DT_B_ was added overnight after cell attachment. After 48 h, Caspase-Glo 3/7 Assay (Promega) regent was added to each well and luminescence was detected using Tecan Safire2 plate reader. For crystal violet assays, cells were treated as described above and were incubated for 48 h. Following incubation cells were harvested and reseeded at low seeding densities in 6-well plates. Colony formation was monitored over 14 days, during which media was replaced every three days. On day 14 colonies were fixed in crystal violet fixing/staining solution (0.05% (g/vol) crystal violet, 1% formaldehyde, 1% (v/v) methanol in PBS. Open source ColonyArea ImageJ plug-in was used for quantitative analysis of the area % covered by the stained colonies^50^. Due to high background from crystal violet staining in SW620 cells, stained wells were dissolved in 10% acetic acid and destained on rocker for 30 min. Absorbance was measured at 590 nm using Tecan Safire2 plate reader.

### Proteome human phospho-kinase array

CRC cell lines were treated as described and washed in 1X PBS. Cells were solubilized using lysis buffer provided by the vendor (R&D Systems) and rocked for 30 min at 4 °C. Suspension was spun for 5 min at 14,000 × *g* and supernatant was collected. Concentration of protein in the collected supernatant fluid determined using the BCA assay (ThermoFisher Scientific, no. 23227). 200 μg of sample lysate was applied to nitrocellulose membranes kinase arrays and incubated overnight at 4 °C. Provided detection antibodies were incubated with specified concentrations as suggested by the supplier. Membrane arrays were acquired using Odyssey Infrared Imaging System (LI-COR Biosciences) and quantified by densitometry using NIH ImageJ software. Values from densitometry analysis were normalized to HSP60 control. Normalized value was then converted to Log_2_ fold change and plotted on heatmap using Graphpad Prism 8.

### Cell cycle flow cytometry

CRC cell lines were treated as described above. After 24 h of treatment, cells were collected from medium, washed with 1X PBS, and released from well with Trypsin–EDTA (0.25%), phenol red (Invitrogen). Harvested cells were centrifuged at 700 × *g* for 5 min. Cells were washed twice in PBS and spun down at 700 × *g* for 5 min. PBS was removed and cells were resuspended in 600 μL of ice-cold PBS. Cell were permeabilized with addition of 1.4 mL of ice-cold ethanol slowly and incubated overnight at -20 °C. Following two washes with PBS (centrifuged at 700 × *g* for 5 min), cells were stained in 200 μL PI staining solution (0.1% Triton X-100, 50 μg propidium iodide (BioLegend), 0.2 mg/mL RNase) for 30 min. Samples were analyzed on BD LSR Fortessa 1 Analyzer. At least 10,000 events were collected for each sample. Single cell populations were viewed and gated on cyanine-3 area (Cy3-A) versus cyanine-3 width (Cy3-W) channels, to eliminate doublet events. ModFit LT Software (Version 5) was used for cell cycle analysis.

## Supplementary Information

Supplementary Information.
