# Supplementary material for: RAS specific protease induces irreversible growth arrest via p27 in several KRAS mutant colorectal cancer cell lines
Source: Sci Rep. 2021 Sep 9;11:17925. doi: 10.1038/s41598-021-97422-0 (PMC8429734; doi:10.1038/s41598-021-97422-0)
Supplement: Supplementary file 1 — Supplementary Information. [file 41598_2021_97422_MOESM1_ESM.pdf]

RAS specific protease Induces Irreversible Growth Arrest via p27 in several KRAS Mutant Colorectal Cancer cell lines

Caleb K. Stubbs, Marco Biancucci*, Vania Vidimar*, Karla J. F. Satchell†

Department of Microbiology and Immunology, Northwestern University, Feinberg School of Medicine, Chicago, Illinois.

*Present addresses: M.B., GSK Vaccines, Rockville, MD 20850, USA; V.V., Merck Research Laboratories, Boston, MA 02115

†Corresponding author: Karla Satchell, Northwestern University, Feinberg School of Medicine, Chicago, IL 60611, USA. Email: k-satchell@northwestern.edu

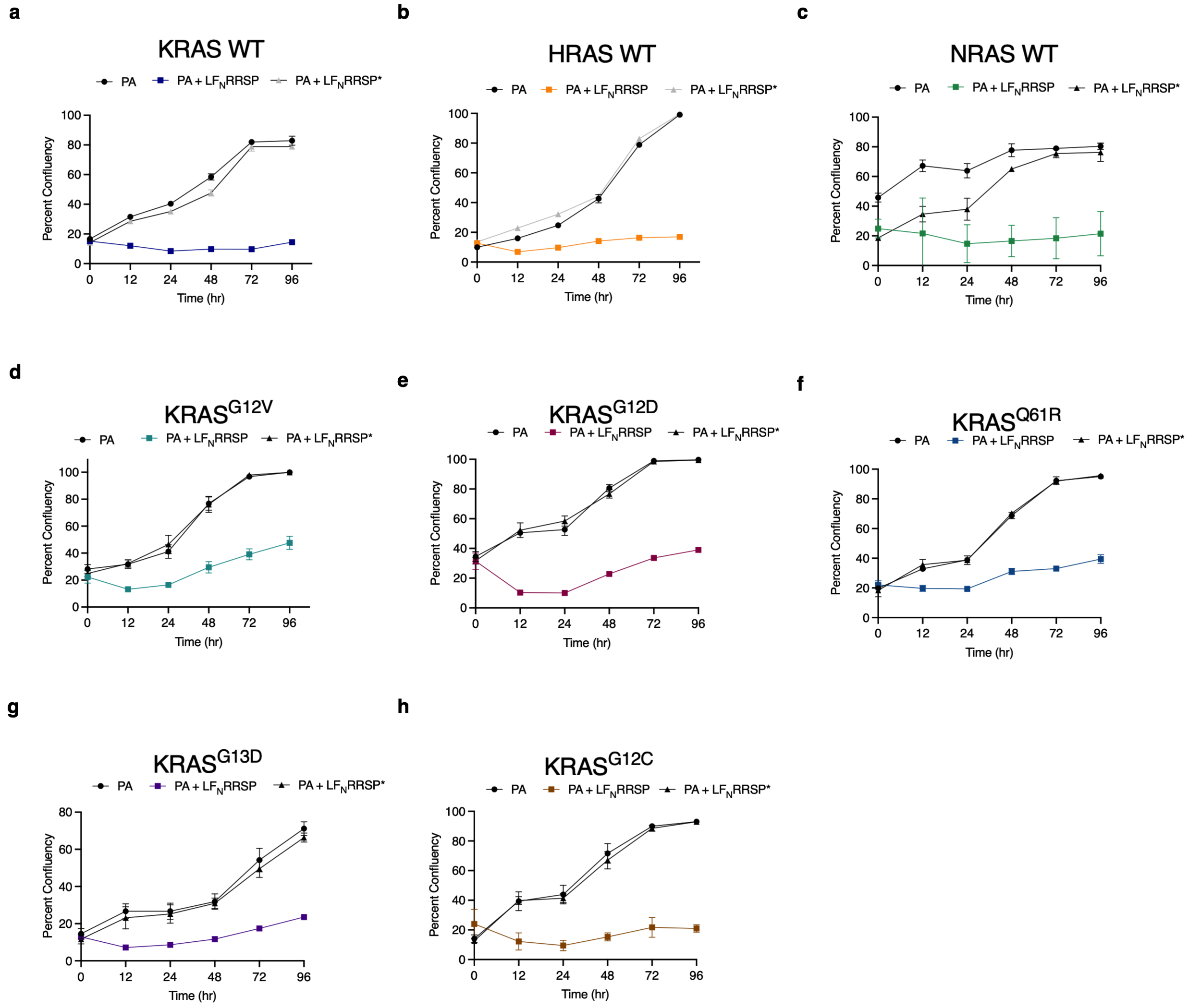

Supplementary Figure 1. Growth inhibition of RAS-less MEFs cell lines. (**A-H**) Growth inhibition observed in of RAS-less MEF cell lines at indicted timepoints following treatment with PA alone or in combination with LF_NRRSP or LF_NRRSP*; *n* = 3. Results are expressed as ± SEM.

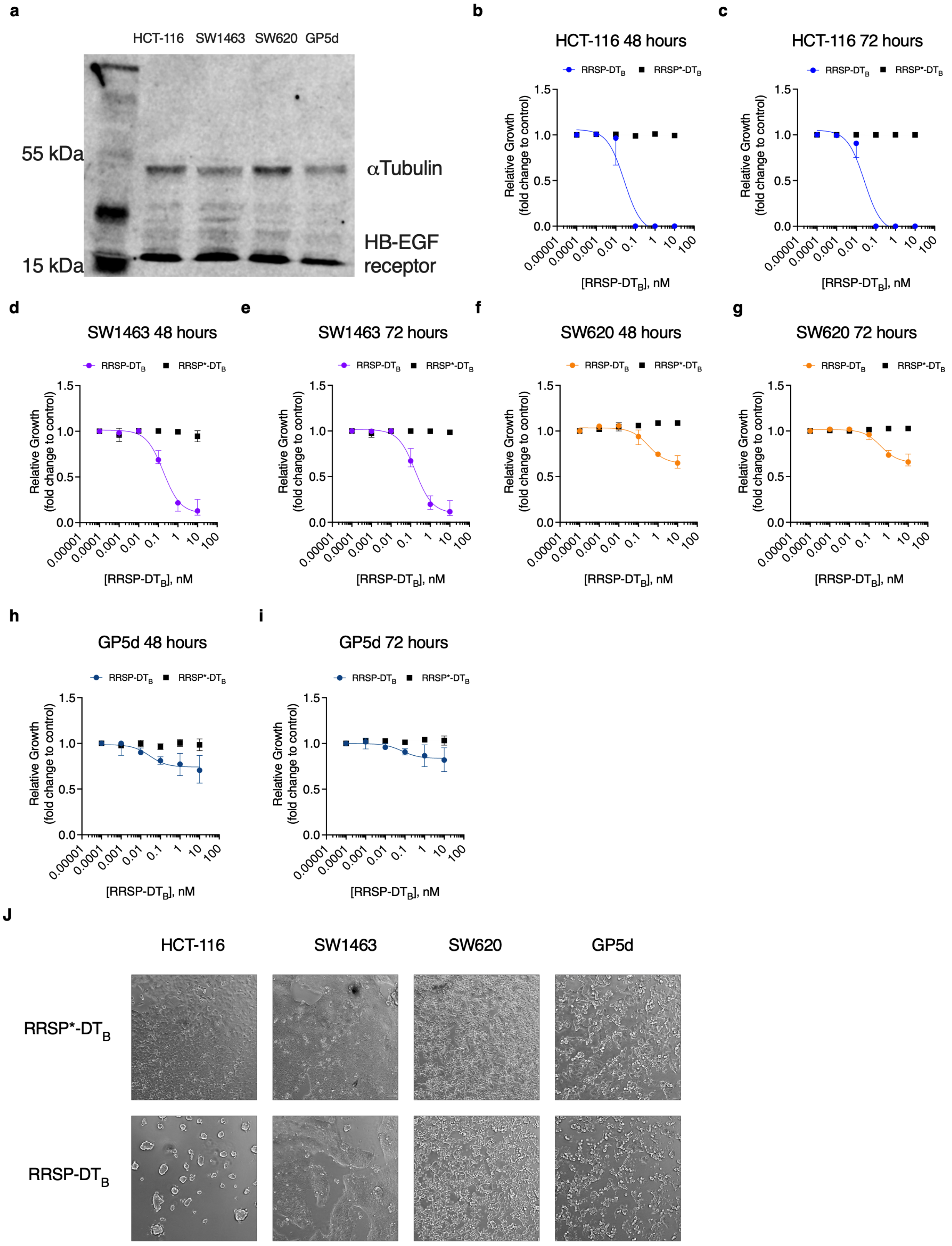
a
HCT-116 SW1463 SW620 GP5d
55 kDa
15 kDa
αTubulin
HB-EGF receptor
b
HCT-116 48 hours
c
HCT-116 72 hours
d
SW1463 48 hours
e
SW1463 72 hours
f
SW620 48 hours
g
SW620 72 hours
h
GP5d 48 hours
i
GP5d 72 hours
RRSP-DTB
RRSP*-DTB
Relative Growth (fold change to control)
[RRSP-DTB], nM
0.00001 0.0001 0.001 0.01 0.1 1 10 100
0.0 0.5 1.0 1.5
J
HCT-116 SW1463 SW620 GP5d
RRSP*-DTB
RRSP-DTB

Supplementary Figure 2. Growth inhibition in RRSP-DT_B treated CRC cell lines. (**A**). Western blot analysis of HB-EGF receptor (DT receptor) from untreated CRC cell line lysates. Vinculin was used as loading control. (**B**-**I**) Cell growth over time was monitored by time lapse video microscopy and quantified using Nikon Elements to calculate cell confluency. Fitted dose response curve of RRSP-DT_B in CRC cell lines show relative confluency compared to PBS values after 48 and 72 hours. Results are displayed as mean ± SEM, *n* = 4. (**J**) Representative brightfield images of CRC cell lines treated with either RRSP-DT_B or RRSP*-DT_B (0.1 nM) after 24 hours.

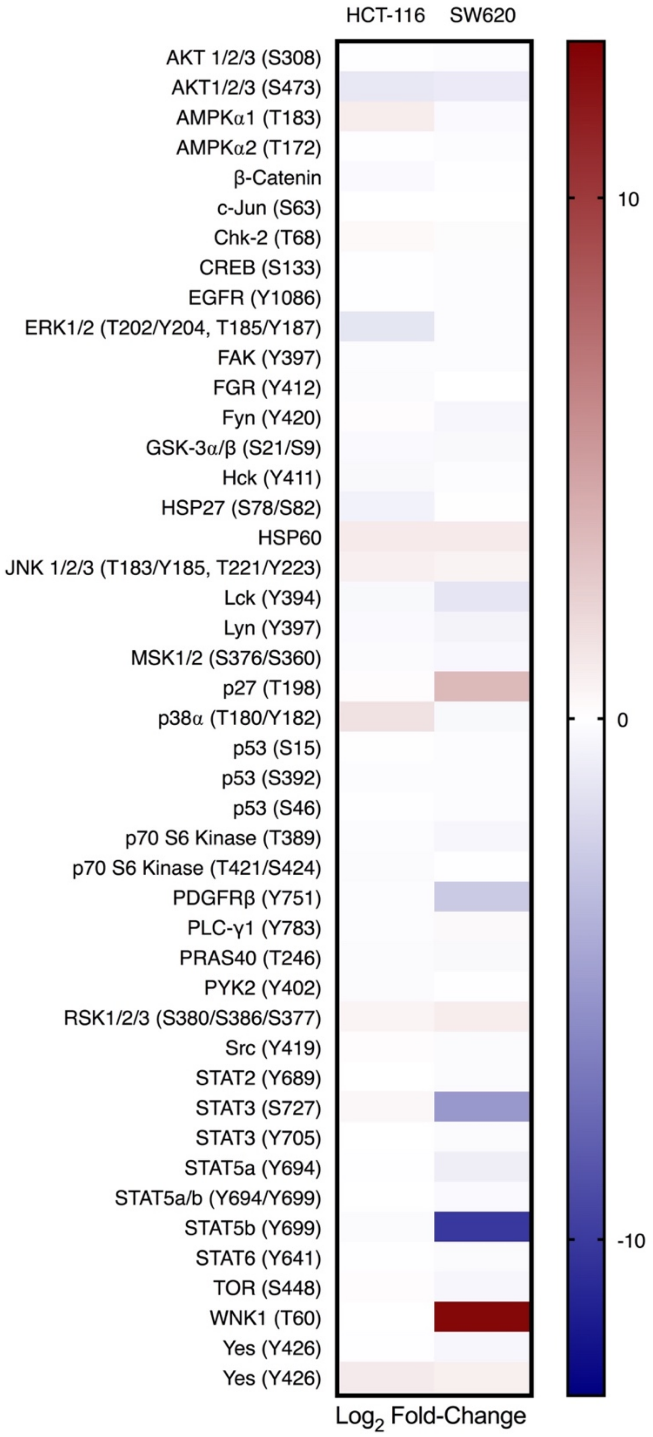

Supplementary Figure 3. Heatmap of human phospho-kinase array in HCT-116 and SW620 cells treated RRSP-DT_B treated samples. Densitometric analysis of phospho-kinase array depicted through a heatmap of relative phosphorylated proteins levels in response to 10 nM RRSP-DT_B compared to PBS control in HCT-116 and SW620 cells after 24 hr.

a

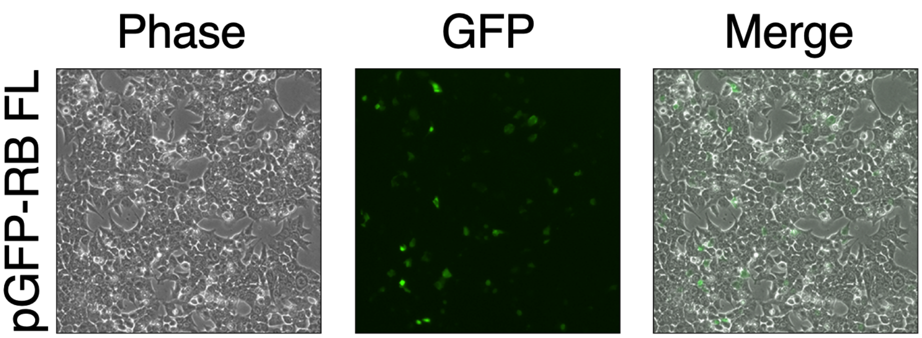

b

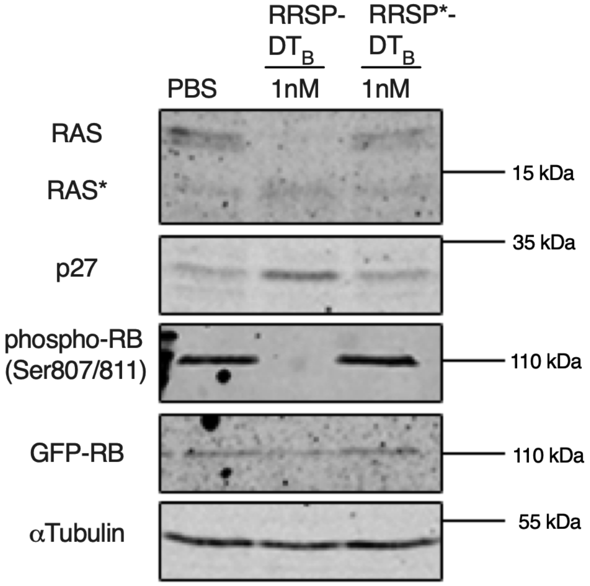

c

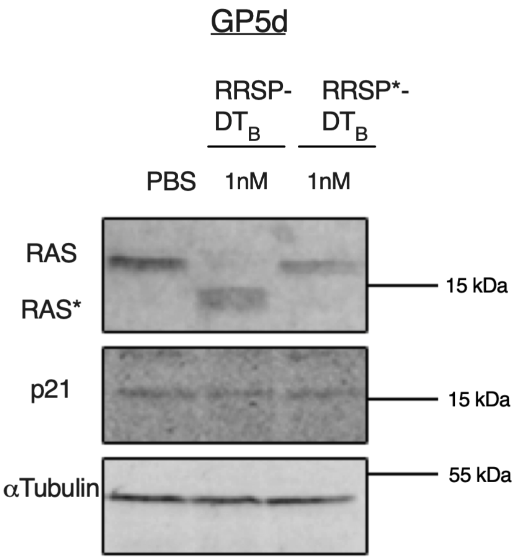

Supplementary Figure 4. Total RB expression in CRC cell lines. (**A**) Representative images of HCT-116 cells transfected with pGFP-RB after 24 hr (**B**) Western blot analysis of HCT-116 cells transfected with GFP tagged RB following treatment with either PBS, RRSP-DT_B or RRSP*-DT_B after 24 hours. Total RB was detected using anti-GFP primary antibody. αTubulin was used as a gel loading control. (**C**) Western blot analysis of p21 levels in GP5d cell treated with either PBS, RRSP-DT_B or RRSP*-DT_B after 24 hr.

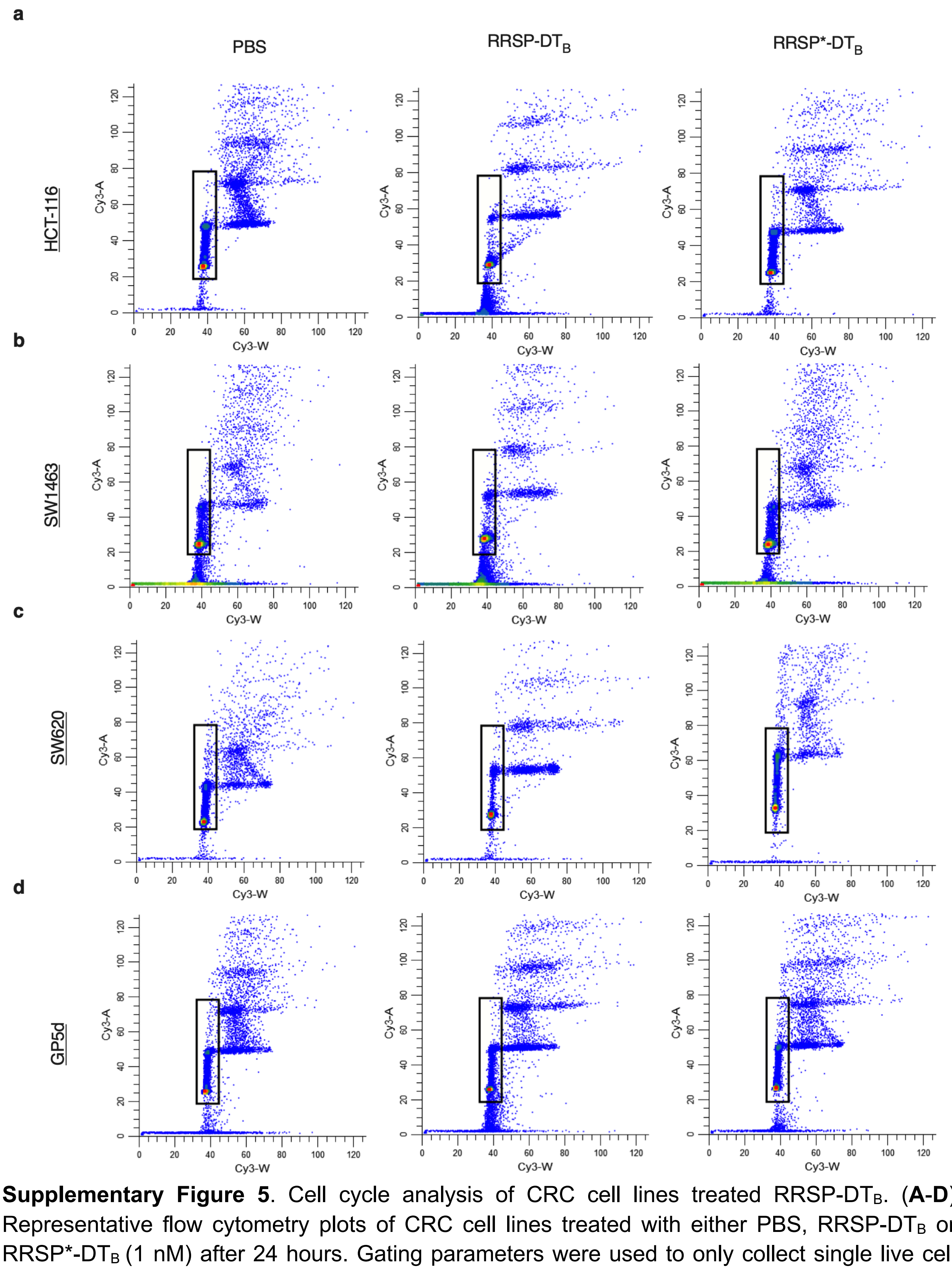

Supplementary Figure 5. Cell cycle analysis of CRC cell lines treated RRSP-DT$_B$. (**A-D**) Representative flow cytometry plots of CRC cell lines treated with either PBS, RRSP-DT$_B$ or RRSP*-DT$_B$ (1 nM) after 24 hours. Gating parameters were used to only collect single live cell populations.

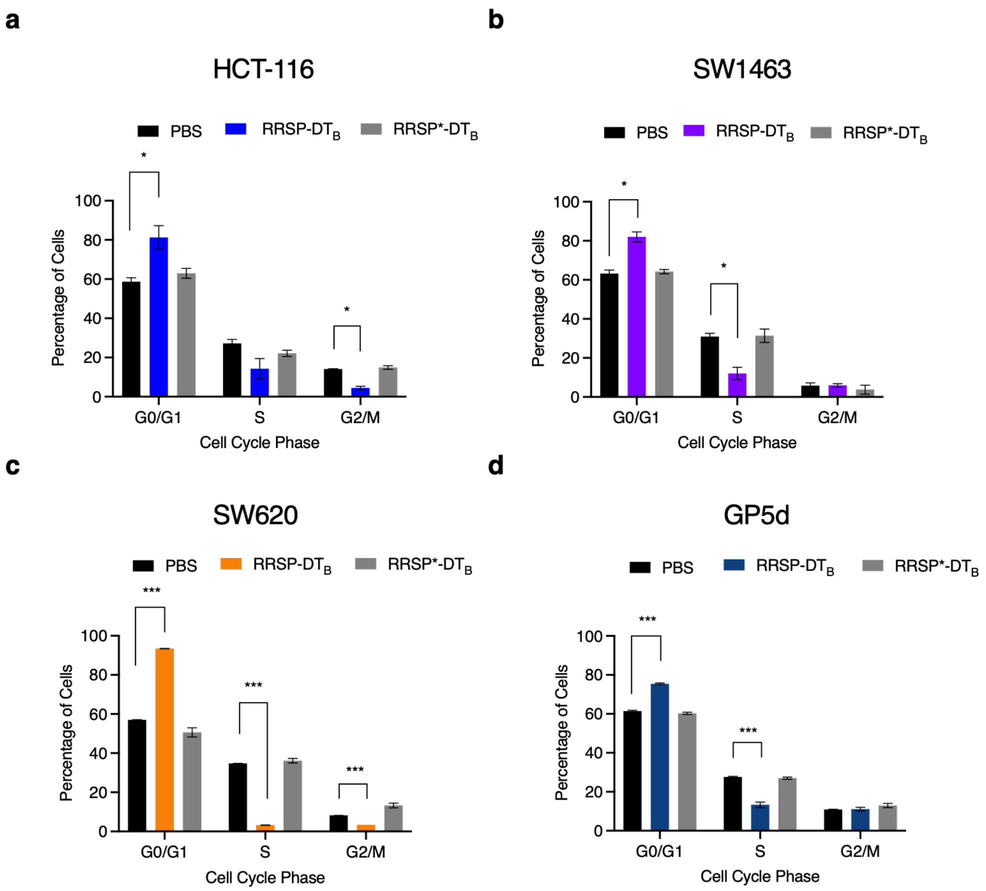

Supplementary Figure 6. Cell cycle analysis of RRSP-DT$_B$ treated CRC cell lines. (**A-D**) Quantitative analysis of cell cycle analysis of CRC cell lines treated with PBS, 1 nM RRSP-DT$_B$ or 1 nM RRSP*-DT$_B$ for 24 hours; *n* = 3. Results are expressed as means ± SEM of three independent experiments (*P< 0.05, **P<0.01, ****P<0.0001 versus PA control as determined through one-way ANOVA followed by Dunnett's multiple comparison test).

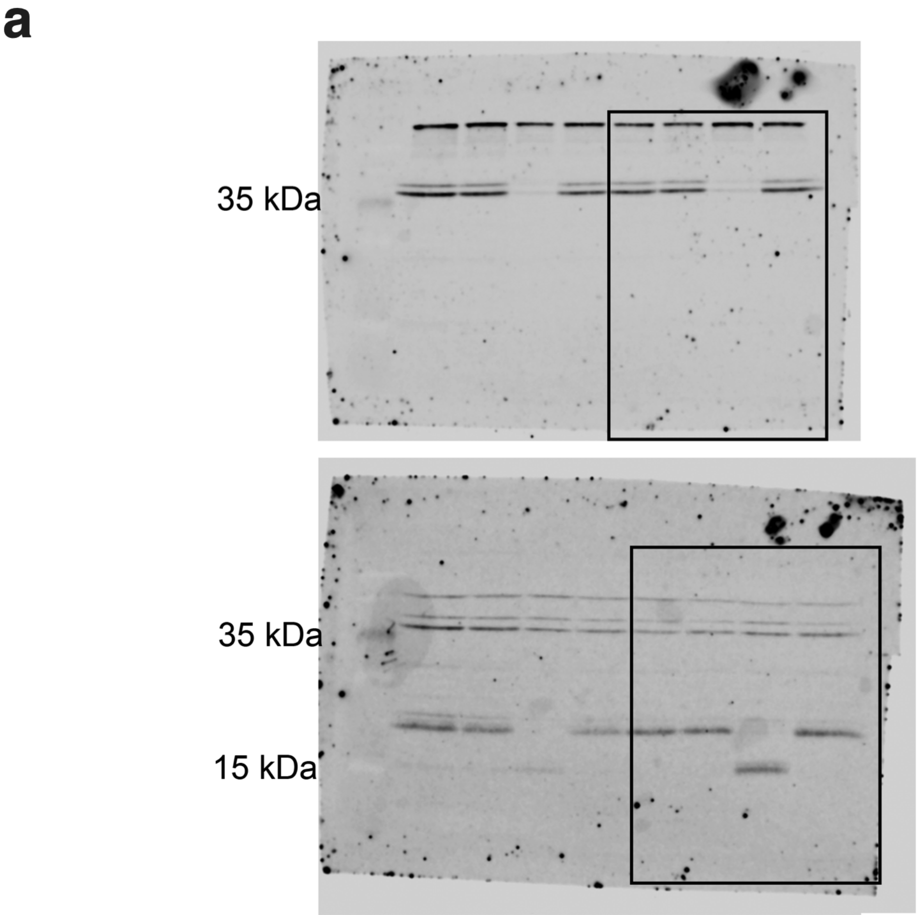

Supplementary Figure 7. Uncropped immunoblots for Fig 1A (A). Lanes from main figure are shown within rectangle. Membrane was stripped and reprobed for target proteins sharing similar molecular weights.

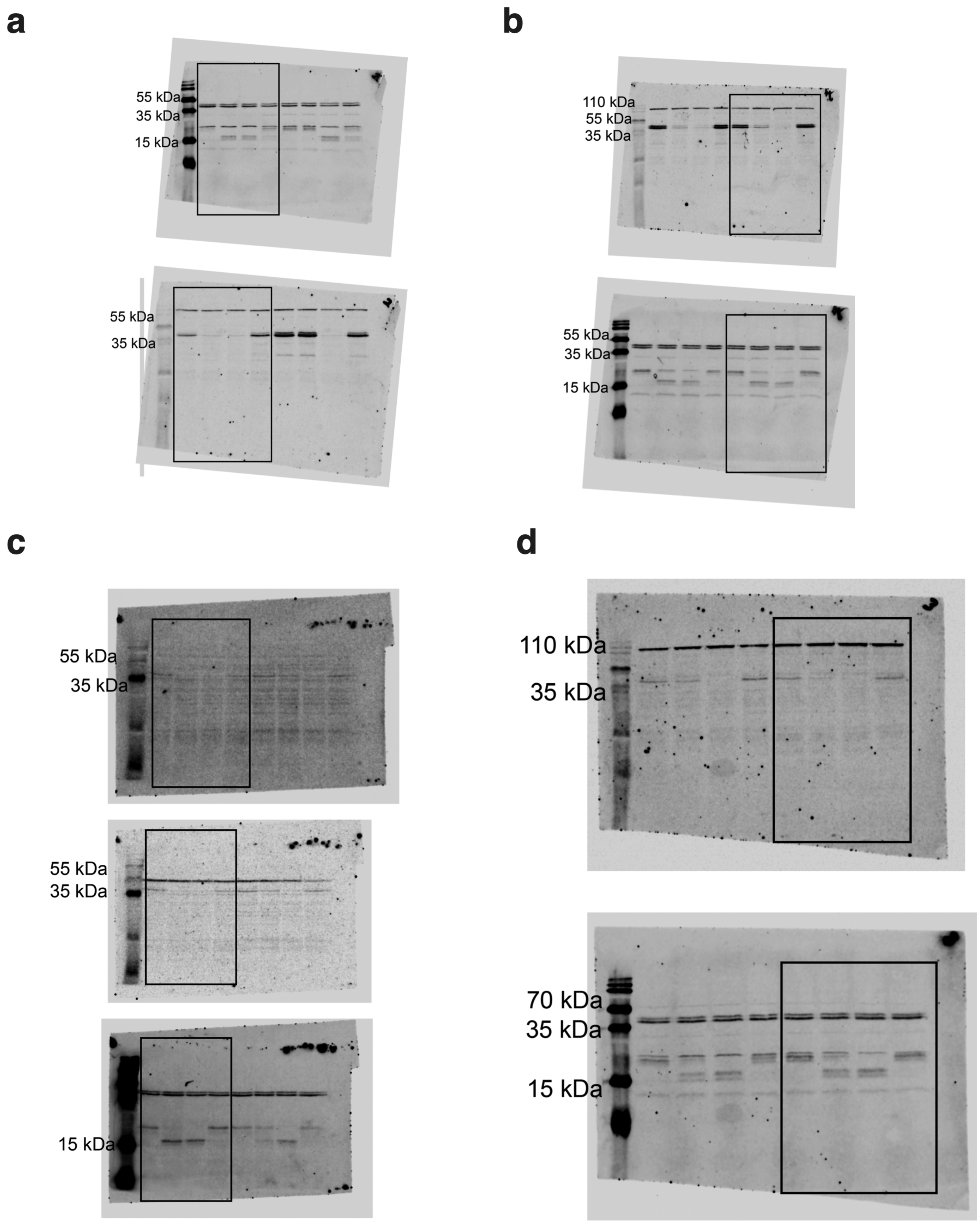
a
55 kDa
35 kDa
15 kDa
55 kDa
35 kDa
b
110 kDa
55 kDa
35 kDa
55 kDa
35 kDa
15 kDa
c
55 kDa
35 kDa
55 kDa
35 kDa
15 kDa
d
110 kDa
35 kDa
70 kDa
35 kDa
15 kDa

Supplementary Figure 8. Uncropped immunoblots for Fig 2F (A-D); (A) HCT-116 (B) SW1463 (C) SW620 (D) GP5d. Lanes from main figure are shown within rectangle. Membrane was stripped and reprobed for target proteins sharing similar molecular weights.

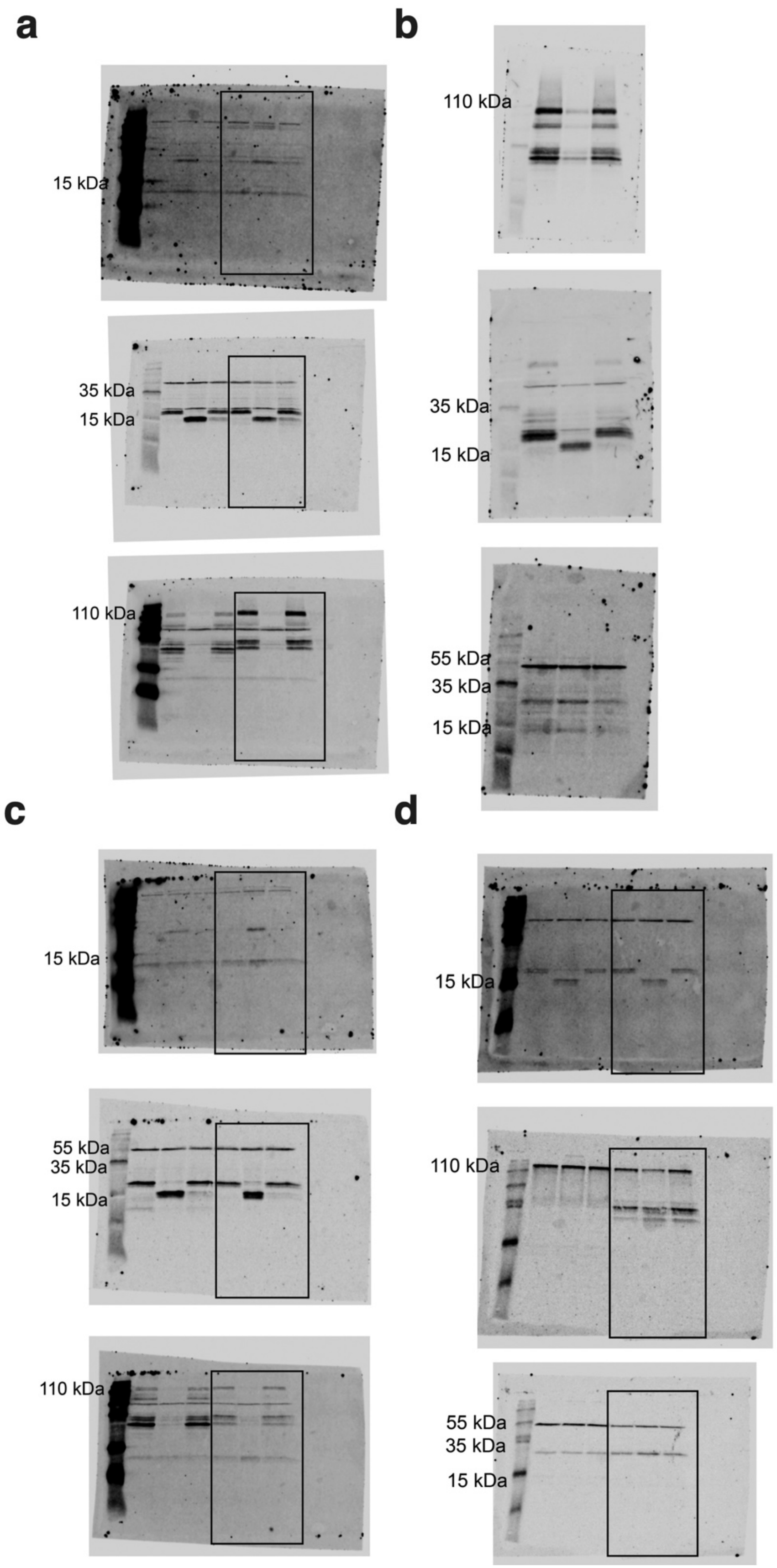
a
15 kDa
35 kDa
15 kDa
110 kDa
b
110 kDa
35 kDa
15 kDa
55 kDa
35 kDa
15 kDa
c
15 kDa
55 kDa
35 kDa
15 kDa
110 kDa
d
15 kDa
110 kDa
55 kDa
35 kDa
15 kDa

Supplementary Figure 9. Uncropped immunoblots for Fig 4C (A-D); (A) HCT-116 (B) SW1463 (C) SW620 (D) GP5d. Lanes from main figure are shown within rectangle. Membrane was stripped and reprobed for target proteins sharing similar molecular weights.

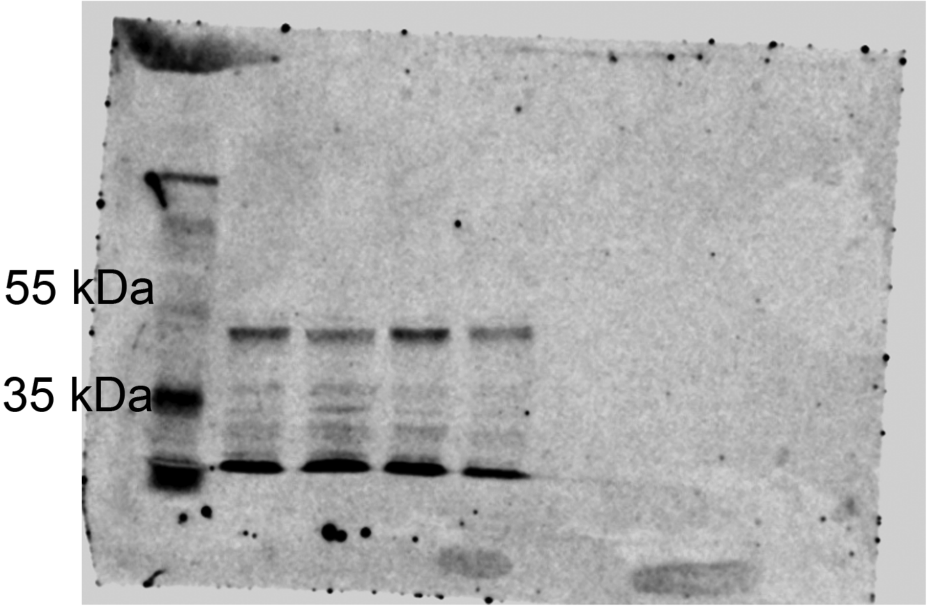

Supplementary Figure 10. Uncropped immunoblots for Supplementary Fig 2A. Lane 2: HCT-116. Lane 3: SW1463. Lane 4: SW620. Lane 5: GP5d.

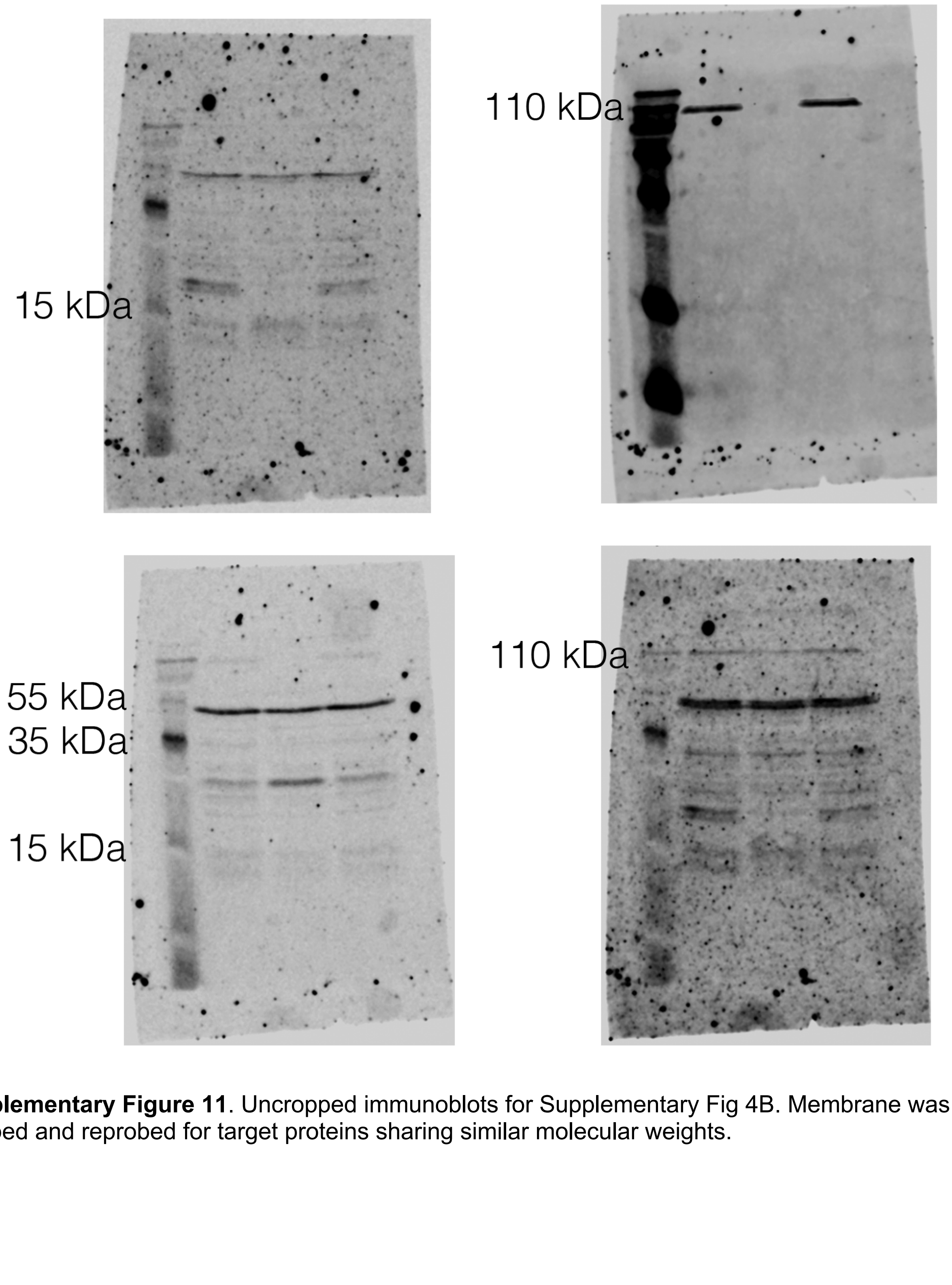

Supplementary Figure 11. Uncropped immunoblots for Supplementary Fig 4B. Membrane was stripped and reprobed for target proteins sharing similar molecular weights.

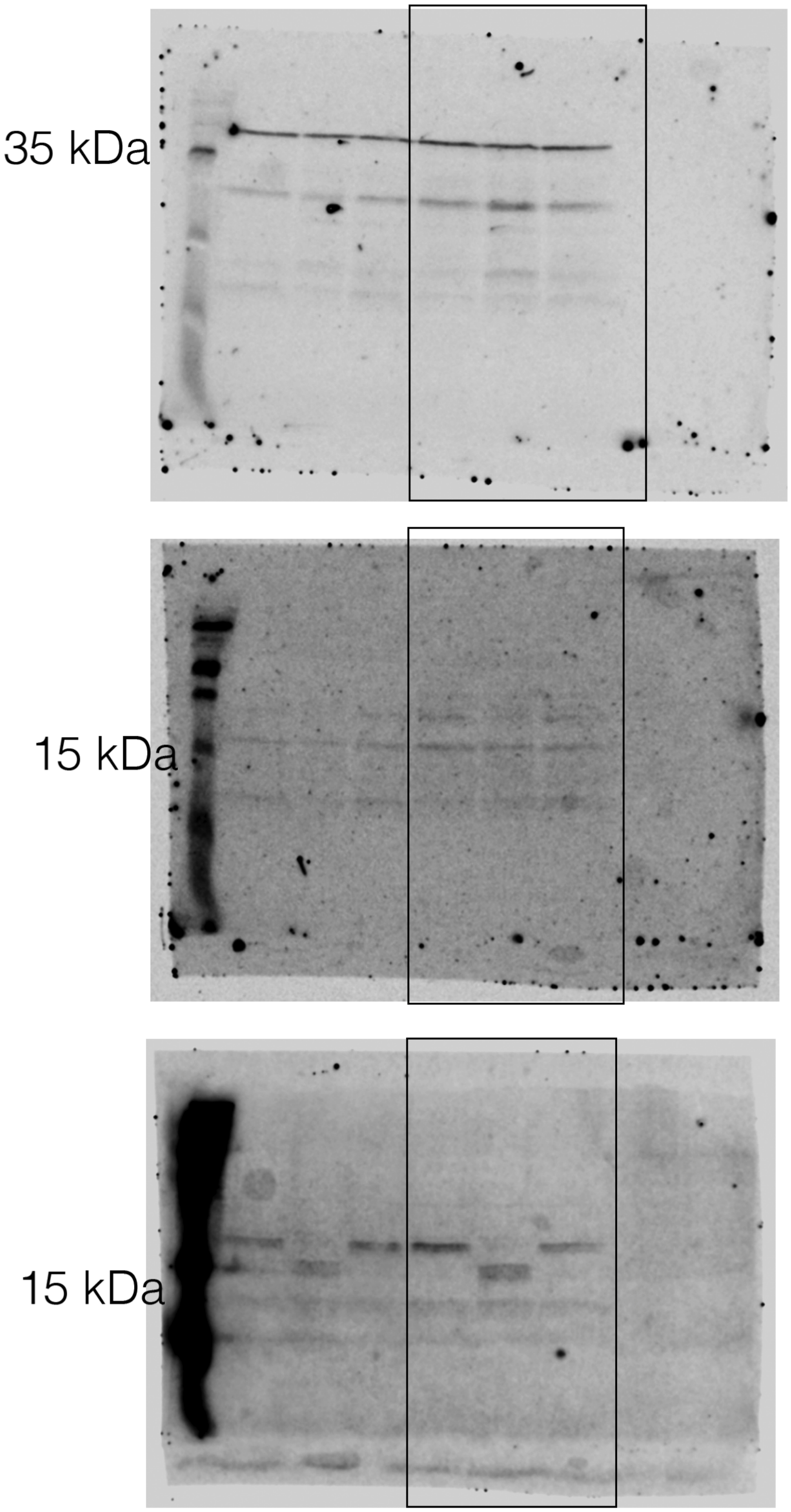
35 kDa
15 kDa
15 kDa

Supplementary Figure 12. Uncropped immunoblots for Supplementary Fig 4C. Lanes from main figure are shown within rectangle. Membrane was stripped and reprobed for target proteins sharing similar molecular weights.
